# Focus and Insights into the Synthetic Biology-Mediated Chassis of Economically Important Fungi for the Production of High-Value Metabolites

**DOI:** 10.3390/microorganisms11051141

**Published:** 2023-04-27

**Authors:** Pragya Tiwari, Laurent Dufossé

**Affiliations:** 1 Department of Biotechnology, Yeungnam University, Gyeongsan 38541, Republic of Korea; pragyamita02@gmail.com; 2 Chemistry and Biotechnology of Natural Products, CHEMBIOPRO, Université de La Réunion, ESIROI Agroalimentaire, 15 Avenue René Cassin, F-97490 Saint-Denis, France

**Keywords:** β-lactam antibiotics, functional food, fungal chassis, genome editing, CRISPR-Cas, *Saccharomyces cerevisiae*

## Abstract

Substantial progress has been achieved and knowledge gaps addressed in synthetic biology-mediated engineering of biological organisms to produce high-value metabolites. Bio-based products from fungi are extensively explored in the present era, attributed to their emerging importance in the industrial sector, healthcare, and food applications. The edible group of fungi and multiple fungal strains defines attractive biological resources for high-value metabolites comprising food additives, pigments, dyes, industrial chemicals, and antibiotics, including other compounds. In this direction, synthetic biology-mediated genetic chassis of fungal strains to enhance/add value to novel chemical entities of biological origin is opening new avenues in fungal biotechnology. While substantial success has been achieved in the genetic manipulation of economically viable fungi (including *Saccharomyces cerevisiae*) in the production of metabolites of socio-economic relevance, knowledge gaps/obstacles in fungal biology and engineering need to be remedied for complete exploitation of valuable fungal strains. Herein, the thematic article discusses the novel attributes of bio-based products from fungi and the creation of high-value engineered fungal strains to promote yield, bio-functionality, and value-addition of the metabolites of socio-economic value. Efforts have been made to discuss the existing limitations in fungal chassis and how the advances in synthetic biology provide a plausible solution.

## 1. Introduction

The increasing prevalence of chronic diseases including neurodegenerative, diabetes, and cardiovascular diseases can be addressed by adopting a healthy lifestyle and a balanced diet. Different kinds of less/fat-free diets, low carbohydrate diets [1], and soy-based diets [2] including others are gaining importance and are adopted for good health, together with increased usage of functional food, nutraceuticals, and drugs to delay aging and promote well-being [3,4,5,6]. Microorganisms define a key platform to produce different kinds of high-value metabolites/food products for thousands of years, and have garnered attention in commercial initiatives, towards the bio-based production of metabolites for value addition [7]. Several decades have witnessed the consumption of fungi as functional food, majorly including edible mushrooms (*Ascomycota* and *Basidiomycota*), regarded as edible delicacies and key nutritional sources, across the globe. Although several yeasts (*Pichia pastoris*, *Saccharomyces cerevisiae*, and *Yarrowia lipolytica*) and multiple fungal strains have been industrially employed to produce high-value metabolites, filamentous fungi demonstrate key properties, including presence in solid-state cultures, metabolite production, and polymers degradation, and thereby, prospects in commercial applications [8,9]. The presence of natural products in fungal species provides a defense against predators, harmful UV radiation, and competitive microbes, ensuring their survival in the ecological niche, while broad-spectrum functions prove beneficial for human health [10]. In the present era, several fungal species (including non-*Basidiomycota* species) are being explored and commercialized as nutraceuticals/supplements, and functional foods attributed to multiple pharmacological properties [11,12], and some well-known examples include *Fusarium*, *Aspergillus,* and *Penicillium* sp. The mycelia of some fungal species comprise healthy lipids, dietary fiber, vitamins, etc., and the consumption of these components defines key health advantages [13,14]. Certain filamentous fungi are used as an alternative protein source (due to higher protein contents) [13] while other species are defined as good sources of food flavors, probiotics, and healthy food lipids, respectively. Recent advances in scientific interventions (e.g., 3D printing in food) are widely employed in improving food product shapes, colors, sizes, and textures [15].

Present decade has witnessed research initiatives on filamentous fungi yielding high-value substances of socio-economic significance, including citrate from *Aspergillus niger* [16], penicillin from *Penicillium rubens* [17], pigments [18], pharmaceuticals (lovastatin and cyclosporine) [19,20], industrially important enzymes from *A. oryzae* and *A. niger* [21], lycopene (carotenoids) from *Blakeslea trispora* [22], organic acids (itaconic acid) and secondary metabolites (lovastatin) from *A. terreus* [23,24,25], β-lactam antibiotics (cephalosporins) from *Acremonium chrysogenum* [26], riboflavin (vitamin B2) from *Ashbya gossypii* [27], β galactosidase from *Trichoderma* sp. [28], cosmetics ingredients, namely, kojic acid [29], fortified nutraceuticals lipase (lipopan F, folate) from *Rhizopus oryzae* [30], and *S. cerevisiae* [31], polyunsaturated fatty acid (arachidonic acid) from *Mortierella alpina* [32], probiotics (whole cell) of *Saccharomyces boulardii* [33], and fibrino (geno) lytic enzymes from *Penicillium* sp. [8], including others.

In recent times, the production of food products from microbial sources has witnessed a tremendous upsurge, attributed to the nutritional content and health benefits of high-value compounds. Microbial biosynthesis not only improves the nutritional content/quality of the food, but it also improves the bio-functionality of the food products via imparting beneficial properties including antimicrobial action, peptide production, probiotic properties, removal of antinutritive substances, and fibrinolytic activity, among others [34,35]. A variety of food products/ingredients have been produced by fungi including food pigments, enzymes, nutraceuticals, and pharmaceuticals, among others [36,37]. Functional food represents an alternative means to decrease the likelihood of chronic diseases by optimizing or addressing any defects in metabolic processes, thereby promoting quality of life. Edible mushrooms are cultivated worldwide, constituting a high protein source in balanced diets [38]. Edible mushrooms comprise a key protein source, with good concentrations of unsaturated fats, fibers, minerals, vitamins, etc. [39]. Nutritional benefits of fungal species, amino acid profiles, and high protein content (some filamentous species) comprise distinct benefits [13,40], while other fungal species are valued for the presence of healthy lipids, probiotics, flavors, etc. [41]. Oligosaccharides produced from copropilous fungi are gaining attention as functional food, comprising significant health-promoting properties [42]. The low molecular weight carbohydrates include xylooligosaccharide, inulooligosaccharide, and fructooligosaccharide and are used as substitutes for low cariogenic sugar in the diet. In this direction, key studies discussed the biological activities of polysaccharides isolated from edible fungi and comprise: antioxidant polysaccharides from *Tremella* and their free-radical scavenging effect, and polysaccharides from *Morchella esculenta* improve the activity of antioxidant enzymes [43]; antitumor polysaccharides from *Agaricus bisporus* controlling cancer cell lines growth [44]; anti-aging polysaccharides from *Tremella* that relieve epidermal bleeding and inflammation [45]; and immunomodulatory polysaccharides from *Ganoderma* that promote the immune function [46], among others. Furthermore, fungal species have been extensively documented to be an invaluable source of functional foods and nutraceuticals and marketed as *Ganoderma* capsules (for increased immunity) [47], Lentinan from *Lentiana edodes* (as chemotherapy adjuvant) [48], Hanqi edible mushroom products from *Coprinus comatus*, and *A. camphorata* (which reduce high blood pressure) [49], pork sausage from *Pleaurotus eryngii *(food ingredient) [50], Canned ground ham from Winter mushroom (a substitute of synthetic nitrite) [51], and Pasta from *L. edodes* (nutritional supplement) [52], among other examples.

**Figure 1:**
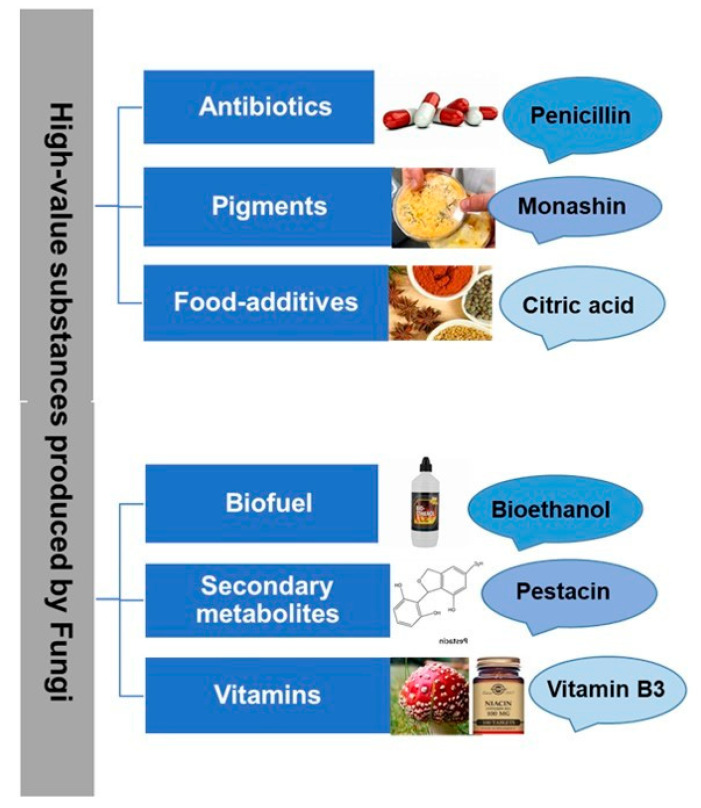
Schematic outline of high-value substances of socio-economic significance produced from fungi.

Several fungi culture strategies, including solid-state fermentation (SSF) and liquid-state fermentation (LSF), increase the production of fungal biomass, facilitating an easy recovery and increased metabolite production [53]. Furthermore, biotechnological interventions have facilitated the monitoring of variables in edible fungi cultivation, extraction of bioactive constituents, redefining the commercialization of functional food from fungi. Figure 1 provides a schematic outline of high-value substances of socio-economic significance produced from fungi.

Fungal biotechnology aims to define and harness the metabolic functions of filamentous fungi of potential interest and their commercialization in diverse industries, and the health sector [54]. The branch of fungal biotechnology has increasingly tried to promote a bio-based economy and develop alternative biological resources to address the growing global food demands [35,36]. Studies are increasingly investigating the economic potential of fungal species, via metabolic pathway elucidation and optimization of fungal strains, toward the development of efficient biofactories [55,56]. However, multiple bottlenecks/knowledge gaps exist in the complete exploitation of beneficial fungal strains via metabolic engineering. The fungal species *Penicillium chrysogenum*, *Aspergillus terreus*, *Trichoderma reesei*, and *Thermothelomyces* thermophiles are selected for genetic manipulations attributed to their biotechnological potentials [21,57]. Particularly, *A. niger* is extensively used in diverse industrial applications and harnessed to produce food ingredients, metabolites, vegan leather, vitamins, etc. [21]. Besides *A. niger*, many other filamentous strains are used nowadays, in diverse commercial applications, including biofuel, textile, food, pharma, and chemical industries.

While substantial progress has been made in engineering *S. cerevisiae*, the metabolic engineering attempts toward the chassis of filamentous fungi are still limited. The synthetic biology-mediated chassis of fungal strains for introducing a desired trait or enhancing the production of high-value metabolites for biotechnology or therapeutics is gaining momentum. Biotechnological advances in genome editing via CRISPR-Cas, metabolic pathway reconstitution, and expression in a heterologous host, and gene knockouts, have yielded translational success, as exemplified by key examples [10,19,58]. We herein provide a comprehensive insight into the current state of the art of metabolic engineering initiatives in fungi towards enhanced production of high-value metabolites and functional foods.

## 2. Nanotechnology and Fungal Biotechnology

The bioactive compounds produced by fungi comprise novel scaffolds and are key bioresources in multi-faceted applications, ranging from bioactive compounds in healthcare to high-value functional food and compounds of commercial/industrial value. Fungal species as a biological platform have the potential for nanoparticle synthesis via extracellular or intracellular enzyme reduction [59]. The prospective application of fungi-synthesized nanoparticles comprises use in medical imaging, in the drug-delivery system, and in cancer treatment, respectively. In addition, nanoparticles from fungi are employed in the management of plant diseases and the production of fungicides in agriculture. The emerging field of nanobiotechnology has opened new avenues in fungal biotechnology, with significant applications in a socioeconomic context [60]. Table 1 discuss representative examples of fungi-mediated nanoparticle biosynthesis and their application in agriculture, healthcare, and industries.

**Table 1:** Discussion of representative examples of fungi-mediated nanoparticles and their application in agriculture, healthcare, and industries.

Nanoparticle	Fungal Species	Socio-Economic Application	Reference
Biomedical and therapeutic applications			
AgNP	*Trichoderma viride*	Combination with antibiotics produces a synergistic effect	[61]
AuNP	*Helmithosporum solani*	Anticancer drug	[62]
AgNP	*Aspergillus fumigatus*	Antiviral against HIV-1	[63]
TiO_2_	*Aspergillus flavus*	Antimicrobial function	[64]
AuNP	*Candida albicans*	Liver cancer detection	[65]
Agricultural applications			
AuNP	*Rhizopus oryzae*	Pesticides	[66]
Ca_3_P_2_O_8_ NP	*Aspergillus tubingensis*	Agriculture	[67]
AuNP	*Fusarium semitectum*	Optoelectronics	[68]
FeCl_3_	*Aspergillus oryzae*	Agriculture	[69]
AgNP	*Rhizopus stolonifer*	-----	[70]
TiO_2_	*Aspergillus flavus TFR7*	Plant nutrient	[71]

## 3. Fungi—An Emerging Biological Platform for Metabolic Engineering

**Figure 2:**
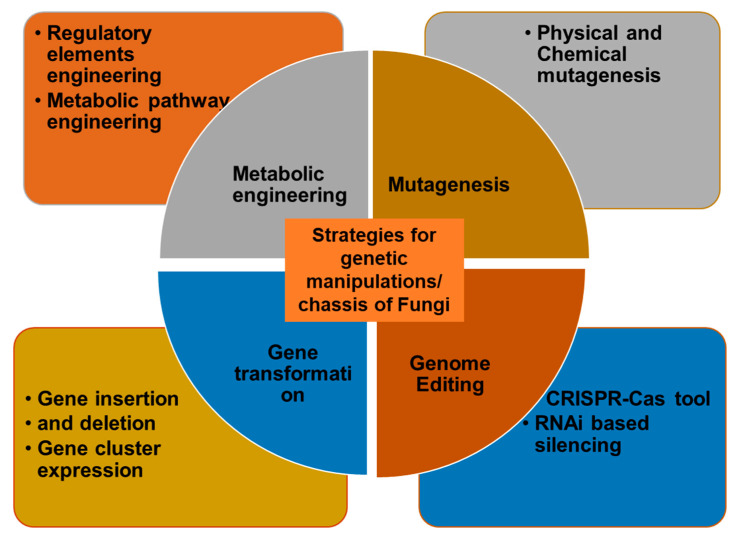
Genetic manipulation strategies for enhanced production of genetically and metabolically modified food products.

The branch of metabolic engineering aims to modify a biological organism to modulate its metabolism. Genetic manipulation is commonly employed in yeast, plants, or bacteria for enhanced production of metabolites, demonstrating socio-economic attributes [72,73] (Figure 2). The tremendous prospects of microorganisms in the biotechnological sector cannot be unseen, with non-model organisms being increasingly explored for human welfare [74,75]. The key aims of metabolic engineering comprise biological strain optimization for enhanced production of high-value substances, yield enhancement, decreased by-product formation, defining a broad substrate range, and improving process efficiency [35,76]. This is achieved by the transfer of enzymes or pathway reconstitution in a heterologous system, to achieve maximum results. Other gene editing methods, namely, DNA recombinant technology, CRISPR/Cas9, and RNA interference, alone or in combination, facilitate the production of value-added compounds including flavonoids, terpenoids, non-ribosomal peptides, alkaloids, biofuels, polymers, and enzymes, and others [77]. In this direction, attempts towards metabolic engineering of biological organisms should focus on the optimization of bioprocess technologies and parameters to obtain the yield of the desired products.

In the past decades, *S. cerevisiae* has been extensively studied as the production platform for metabolites of socio-economic value. Biotechnological interventions have been carried out in yeast including metabolic engineering for fatty acid-derived n-butanol (biofuel) production [78,79], *A. niger* for citric acid and galactaric acid (organic acid) production [80,81], *Y. lipolytica* for omega-3 eicosapentaenoic acid production [82], *Blakeslea tripora* for lycopene (carotenoid) production [22], and other key studies as discussed (Table 2).

**Table 2:** Synthetic biology-mediated engineering of economically viable fungal and yeast strains to produce high-value compounds.

Fungal Strain	High-Value Compounds	Strategy for Metabolic Engineering	Research Outcome	Reference
*S. cerevisiae*	Naringenin (Flavanones)	Genetic manipulation for SmCHS2 expression in a heterologous system	Increased production of naringenin (648.63 mg/L)	[83]
*S. cerevisiae*	Rubusoside and Rebaudiosides	Construction of de novo Rubusoside biosynthetic pathway, removal of rate-limiting steps, metabolic model-based prediction of engineerable targets	Improved production of rubusoside (1368.6 mg/L) and rebaudioside (132.7 mg/L)	[84]
*S. cerevisiae*	Lycopene (carotenoid)	Genome engineering for lycopene pathway optimization and increase in acetyl-CoA pool	Enhanced production of lycopene (56 mg/g)	[85]
*S. cerevisiae*	Eriodictyol (Flavanones)	A cytochrome P450 F3′H from *Gerbera* *hybrida* was functionally expressed in *S. cerevisiae*	Increased production of eriodictyol (200 mg/L)	[86]
*S. cerevisiae*	Fatty acid ethyl esters (FAEEs)	Genetic manipulation of *S. cerevisiae* to utilize glycerol as a substrate for ethanol production	Higher yields of fatty acid ethyl esters	[87]
*S. cerevisiae*	Ethanol	*T. reesei* endoglucanase EGLI and *Saccharomycopsis fibuligera* (β-glucosidase) Bgl1 was introduced into *S. cerevisiae*	Enhanced ethanol production	[88]
*S. cerevisiae*	Fatty acid-derived n-butanol (biofuel)	The silencing of *ADH1*, *ADH4*, *GPD1*, and *GPD2* genes in fungal strain	Increased production of n-butanol (100 mg/L)	[78]
*S. cerevisiae*	Citrate	Deletion of *IDH1* and *IDH2* genes via marker-based homologous recombination	Increased production of citrate	[89]
*S. cerevisiae*	Free fatty acids (FFA)	Pathway reconstitution and optimization (synthetic citrate lyase pathway), Heterologous expression of ATP citrate lyase, and malic enzyme	Increased free fatty acid production (10.4 g/L)	[90]
*S. cerevisiae*	Terpenes	Mevalonic acid (MVA) pathway engineering in *S. cerevisiae*	Enhanced terpene production	[91]
*S. cerevisiae*	Cis, cis-Muconic acid	Pathway engineering (amino acid synthesis) in yeast and conversion of 3-dehydroshikimate (DHS) into cis, cis-muconic acid	Screening for best heterologous genes catalyzing the conversion of DHS to cis–cis muconic acid	[92]
*S. cerevisiae* WRY2	Fatty acids	ATP citrate lyase was introduced and malate synthase was downregulated in the engineered strain	Increased fatty acid production (460 mg/L)	[93]
*S. cerevisiae*	Rosmarinic acid (hydroxycinnamic acid ester)	Metabolic engineering of *S. cerevisiae*	High-level production of rosmarinic acid (5.93 mg/L)	[94]
*S. cerevisiae*	p-hydroxybenzoic acid (PHBA)	Fungal engineering for gene deletion (for negative feedback), overexpression of chorismite lyase (from *E. coli*)	Enhanced production of aromatic compounds	[95]
*S. cerevisiae*	(+)-Valencene and (+)-nootkatone (Sesquiterpenoids)	Combinational engineering comprising promoter change, regulator ROX1 knockout, squalene pathway inhibition, and HMGR overexpression	Enhanced production of β-nootkatol and (+)-nootkatone (170.5 and 45.6 mg/mL)	[96]
*S. cerevisiae*	Fatty acids	Overexpression of *ACC1*, *FAS1*, and *FAS2* genes in the fungal strain	Enhanced fatty acid production	[97]
*S. cerevisiae*	Chlorogenic acid (phenolic compound)	An optimized de novo biosynthetic pathway for CGA was reconstructed in *S. cerevisiae*, a multi-module engineering strategy	Increased production of chlorogenic acid (806.8 mg/L)	[98]
*S. cerevisiae*	Naringenin (Flavanones)	RgTAL (encoding tyrosine ammonia lyase) from *Rhodotorula glutinosa*, Pc4CL (encoding 4-coumaric acid-CoA ligase) from *Petroselinum crispum*, PhCHS from *P. hybrida*, and MsCHI from *Medicago sativa* in *S. cerevisiae*	Increased production of naringenin (29 mg/L)	[99]
*A. niger, S. cerevisiae*	Cheese	Genetic modification with calf rennet gene (chymosin)	Improvement of cheese products	[100]
*Y. lipolytica*	Taxifolin (flavanonols)	Taxifolin biosynthetic pathway expression in *Y. lipolytica*	Increased production of taxifolin (48.1 mg/L)	[101]
*Kluyveromyces lactis*	L-ascorbic acid (vitamin C)	Genetic transformation of *K. lactis* with a plasmid harboring cloned plant genes	Increased production of L-ascorbic acid (30 mg/L)	[102]
*K. lactis*	Bioethanol	Construction and characterization of a null mutant (Δklndi1) in the single gene encoding a mitochondrial alternative internal dehydrogenase	Increased bioethanol production	[103]
*K. lactis*	B-galactosidase	A rational mutagenesis strategy by introducing disulfide bonds in the interface between the enzyme subunits was used	Improvement of β-galactosidase enzyme for high-temperature industrial applications	[104]
*A. niger*	Glucoamylase	*glaA* gene encoding for glucoamylase was expressed in *A. niger* under the control of the tunable Tet-on system, deletion of the *racA* gene in the engineered strain	Enhanced glucoamylase secretion in the engineered strain	[105]
*A. niger*	glucoamylase–glucuronidase (GlaGus) protein	Disruption of ER-associated degradation (ERAD) components, derA, doaA, hrdC, mifA, or mnsA in *A. niger*	High-viable *A. niger* strain, enhanced production of (GlaGus) protein	[106]
*A. niger*	Manganese peroxidase	Heterologous expression of manganese peroxidase (from *Phanerochaete chrysosporium*) in *A. niger*, overexpression of calnexin (chaperone)	Increased production of manganese peroxidase	[107]
*A. niger*	Human α1-proteinase inhibitor (α1-PI) (antitrypsin)	Heterologous expression of Human α1-proteinase inhibitor in *A. niger*, a fusion of α1-PI with glucoamylase G2, transformants screening	biologically active glycosylated r-α1-PI with yields of up to 12 mg/L	[108]
*A. niger*	lignin peroxidase	Heterologous expression of lignin peroxidase (from *Phanerochaete chrysosporium*) in *A. niger* under NOS promoter and terminator	Lignin peroxidase production	[109]
*A. niger*	Secondary metabolites (enniatins)	Heterologous expression of nonribosomal peptide synthetase ESYN (from *F. oxysporum*) under Tet-on hybrid promoter in *A. niger*	Enhanced production of enniatins	[110]
*A. oryzae*	Cellulase activity	Integration of multiple copies of genes encoding cellulases (cellobiohydrolase, endoglucanase, and β-glucosidase) into the fungal genome	Enhanced activity of cellulases	[111]
*A. niger*	Citric acid	The system consists of two expression modules jointly targeted to a defined genomic locus	Expression of the desired gene and its function	[80]
*A. niger*	Galactaric acid (organic acid)	Single- and multiplexed CRISPR approaches for galactarate overproduction	Higher production of galactarate (12 g/L)	[81]
*A. niger*	Vitamin C	Metabolic engineering of the fungal d-galacturonate pathway	A higher titer of vitamin C (170 mg/L)	[112]
*A. niger*	Human granulocyte colony-stimulating factor (G-CSF)	Fusion of G-CSF behind a KEX2 cleavage site downstream of glucoamylase	High yields of protein G-CSF in *A. niger* (5–10 mg/L culture medium)	[113]
*P. pastoris*	(+)-nootkatone	Chimeric strain co-expressing the premnaspirodiene oxygenase *Hyoscyamus muticus* and *A. thaliana* cytochrome P450 reductase. Intracellular production of (+)-valencene by co-expression of valencene synthase	Enhanced production of (+)-nootkatone (208 mg/L)	[114]
*P. pastoris*	Fatty acid alcohols	Metabolic transformation of the fatty acid cell factory	High-level production of fatty acid derivatives (2.0 g/L)	[115]
*P. pastoris*	Malic acid	Metabolic engineering and redistribution of metabolic flux	High-level production of malic acid (2.79 g/L)	[116]
*Yarrowia lipolytica*	β-Carotene (carotenoid)	Optimization of promoter–gene pairs of heterologous *crt* pathway	High yields of β-carotene (6.5 g/L)	[117]
*Trichoderma reesei*	Cellulase	Deletion of the small GTPase rac1 in *T. reesei*	Hyperbranching in *T. reesei* strain enhanced cellulase production	[118]
*Neurospora crassa*	Cellulase	Disruption of gul-1 decreased culture viscosity gul-1 overexpression increased viscosity	Increased cellulase secretion in the engineered strain	[119]
*Y. lipolytica*	Omega-3 eicosapentaenoic acid	Heterologous expression of Δ-9 elongase, a Δ-8 desaturase, a Δ-5 desaturase and a Δ-17 desaturase, transformation in *Y. lipolytica*	High yields of eicosapentaenoic (EPA) acid	[82]
*Mortierella alpina*	Oleic and Linoleic acids	Heterologous expression of the D12-desaturase (from *Coprinopsis cinerea*) in the D6DS activity-defective mutant of *M. alpina*	Enhanced production of oleic and linoleic acids in the engineered strain	[120]
*Xanthophyllomyces dendrorhous*	Zeaxanthin	Overexpression of β-carotene hydrolase and mutagenesis of astaxanthin synthase	Enhanced production of zeaxanthin (0.5 mg/g)	[121]
*T. reesei*	Cellulase	Overexpression of β-glucosidase in *T. reesei* under a strong inducible promoter	Enhanced production of cellulase	[122]
*T. koningii*	Cellulase	RNA interference was used to regulate the expression of the *cre1* gene	Enhanced production of cellulase	[123]
*Blakeslea trispora*	Lycopene (carotenoid)	Fermentation optimization with lycopene cyclase inhibitor	Enhanced production of lycopene (256 mg/L)	[22]
Filamentous fungi	Austinoids (insecticides)	Combinational engineering and rewiring of austinoid pathway	Production of diverse austinoid derivatives	[124]

Among other genetic studies in yeasts, key studies include: *Pichia pastoris* (*Komagataella* spp.) glycosylated recombinant proteins and showed higher protein secretion [125]; *Kluyveromyces lactis* was used for efficient recombinant protein production [126]; *Yarrowia lipolytica* used hydrocarbon source and synthesized fatty products [127]; *S. cerevisiae* was engineered for isoprenoid synthesis [128]; and *P. pastoris* was manipulated for membrane proteins production [129]. In addition, biopharmaceuticals, namely, growth factors, insulin, vaccines, glucagon, etc., have been produced in yeast and approved for commercial use [130].

## 4. Fungal Chassis and Production of Functional Foods

Advances in synthetic biology strategies have contributed significantly to enhancing the production of high-value metabolites from diverse fungal strains. Genetic manipulations and mutagenesis aim to address the yield improvement of the desired metabolites from biological systems [131]. Usually, a biological species with a small genome size is preferred for engineering, since it can be handled with precision. For example, *S. cerevisiae* has efficient integration of DNA at desired regions, facilitating easy biological chassis [132]. With an efficient genome editing tool, CRISPR/Cas9, the design, and development of plasmids have considerably improved, adding versatility to the scope of yeast systems as a biotechnological tool [133,134]. Moreover, synthetic biology strategies are adopted to manipulate *S. cerevisiae* and design promoters, cloning plasmids, methods, etc., to benefit their diverse applications in biotechnology. In this direction, efficient transformation protocols, expression systems, and a range of selection markers have been developed [135,136]. Several research initiatives in the chassis of filamentous fungi have created knock-in, knock-out, desired gene expression, and replacement of genes, in a shorter time. Furthermore, the expression of all genes in a biosynthetic pathway via polycistronic expression cassette, in filamentous fungi, has been achieved and used to produce bioactive metabolites [137]. In another initiative, mutant strains of *Monascus* were developed for enhanced pigment production, monashin via enzyme polyketide synthase mutation [138]. The activation of transcription factors DAF-16/FOXO in *M. purpureus* induced thermal shock protein and superoxide dismutase and improved survival in *C. elegans* [139]. A mutant strain of *M. purpureus* was cultivated in fermented rice extract and produced monapurpureusone (a new azaphylone) and monapurpureusin (a natural product), respectively [140]. The filamentous fungi classified in *Basidiomycetes* biosynthesize natural food colorants such as phenazines, melanins, flavins, azaphilones, and quinones [141]. It is significant to optimize the culture conditions in microbial cultures, for maximum recovery of high-value metabolites, particularly in the production of dyes and pigments originating in plants. The biological production of fungal dyes and pigment and their use define safe and healthy food, compared to synthetic colorants [12]. Recent advances in high-throughput methods, strain improvements, and culture parameters optimization have considerably improved the biomass and production of bioactive compounds (Table 1).

### 4.1. Metabolic Engineering toward Microbial Strain Improvement

High-value metabolites are increasingly identified and isolated from biological organisms, including fungi. However, the amount of these bioactive metabolites is in low concentrations, limiting industrial applications. Moreover, the presence of “cryptic pathways” which are silent and do not produce metabolites is documented and necessitates a requirement of strain improvement approaches [142]. In previous methods, a high-yielding strain was screened among all strains and further improved via mutagenesis and selection [143]. For example, *Penicillium chrysogenum* was screened for penicillin (an antibiotic) production and it produced 100-fold higher titers than the original strain. Further, developments in screening and strain improvement led to industrial production of penicillin (100,000-fold higher than the original strain) [144]. This method is beneficial in the way that no prior knowledge of the microbe’s genetics and the metabolic pathway is required, and the best-screened strain can be employed for metabolite production. Furthermore, multiple strategies can be adopted for the reorientation of metabolic flux toward specific metabolite production, such as improving precursor supply, monitoring gene expression regulation, improving enzyme functions, and metabolic pathway reconstitution in heterologous systems, including others [142].

### 4.2. Mutagenesis Approaches in Fungi

For strain improvement in fungi, different mutagenesis approaches were adopted, using chemical or physical agents. To induce specific or random alterations, low/controlled levels were used, since they may lead to harmful mutations [145], and screening of mutants to locate the desired mutants was attempted. While the chemical agents used are base analogs causing base deamination, mainly GC→AT and AT→GC transitions, and impairment [145], physical mutagens comprise ionizing radiation (γ, X-rays) that cause DNA strand breakage, structural modifications, and ultraviolet radiation, which may cause frameshifts mutations and deletions [145]. In this direction, microwave radiations were also employed for strain improvement [146]. Other mutagens including caffeine lead to frameshift mutations with potent effects in fungi and bacteria, and acridine dyes, and ethidium bromide result in deletions and frameshift mutations [145]. Multiple studies in fungi for strain improvement have employed chemical and physical mutagens in *Trichoderma reesei* Rut C-30, [147], and *Penicillium chrysogenum* [148]. Random mutagenesis was attempted in fungi for the enhanced yield of polyamines [149]. Yang and coworkers [150] employed mutagenesis in *Penicillium oxalicum*, phosphate-solubilizing fungi for improved production of organic acid and phosphate solubilization, respectively, which increased significantly on mutagenesis by radiation. In addition, physical and chemical mutagens were used to produce high-value metabolites from fungi, aimed toward a bio-based economy.

### 4.3. Pathway Engineering in Fungi-Recent Trends and Initiatives

When the precursor supply does not affect product titer, the pathway enzyme expression may result in key outcomes. For example, a high-yielding strain of *P. chrysogenum* BW1890 (with multiple copies of gene clusters) leads to a 64-fold increase in the production of penicillin [151]. The advances in metabolic engineering facilitate the expression of all biosynthetic pathway enzyme(s), providing a solution. Moreover, the expression of single enzymes can be tuned by regulating transcription [152], and protein engineering [153], including other methods. In another key example, penicillin production in *A. nidulans* showed aminoadipyl-cysteinylvaline synthetase (ACVS) as a limiting enzyme, and the gene overexpression for ACVS resulted in enhanced ACVS expression and increased penicillin production [154]. However, gene overexpression for acyltransferase (ACYT), and iso penicillin N synthetase (IPNS), only slightly improved penicillin production [155]. Malla and coworkers [156] studied enhanced doxorubicin production (anticancer polyketide), aided by gene overexpression for glycosyltransferase and deoxysugar biosynthesis [157]. An important consideration suggests monitoring the toxic effects of a metabolite (if any) at higher concentrations, in the case of doxorubicin production, the overexpression of resistance genes was essential [158].

In some organisms, the production of desired metabolites has been improved by altering the regulatory components of a metabolic pathway. For example, in *Streptomyces*, gene clusters encode *Streptomyces* antibiotic regulatory protein (SARP) that positively regulates the production of antibiotics [159]. Moreover, the SARP encoding fredericamycin in *Streptomyces griseus* ATCC 49344 was overexpressed and led to higher antibiotic production in the engineered strain [159]. Furthermore, SARP *MtmR* (mithramycin gene cluster in *Streptomyces argillaceus*) overexpression increased mithramycin titer 16-fold, respectively, and the MTMR-activated actinorhodin-producing pathway when expressed in *S. coelicolor* [160].

### 4.4. Precursor Supply Increase

For all the major classes of natural products, increasing the supply of precursor molecules has been a successful method in both native and heterologous systems. These precursors can be primary metabolites or those derived from primary metabolites. For example, malonyl-CoA comprises a key precursor for polyketide biosynthesis; Ryu and colleagues attempted *S. coelicolor* engineering by overexpression of ACCase genes for enhanced malonyl-CoA production, and the study resulted in enhanced actinorhodin production [161]. Zha and coworkers [162] combined multiple methods for increased malonyl-CoA levels in *E. coli*, including pathway knockouts, gene overexpression, and limiting pathways for malonyl-CoA degradation, resulting in a 15-fold increase in malonyl-CoA [162]. Substantial initiatives in engineering *E. coli* for precursor supply have focused on the heterologous expression of the MVA pathway or its improvement for increased isopentenyl pyrophosphate (IPP) production, a precursor in the generation of terpenoids [163,164]. Research initiatives focusing on MEP pathway engineering have shown that 1-deoxy-D-xylulose-5-phosphate reductase (*dxr*), 1-deoxy-D-xylulose-5-phosphate synthase (*dxs*), and isopentenyl diphosphate isomerase (*idi*) overexpression enhanced production of isoprenoids [165]. In primary metabolism, the shikimate pathway is a key component, generating precursors for the biosynthesis of aromatic amino acids, utilized by several classes of natural products as precursors in the biosynthesis of metabolites. The yield of natural products has been considerably increased via increasing shikimate pathway flux and steps in amino acid biosynthesis [166].

### 4.5. Downregulation/Deletion of Metabolic Pathways

Another prospective approach in this direction is to delete certain genes for pathway silencing so that associated metabolic pathways and their unnecessary intermediates can be avoided. A key example highlights that squalene synthase in yeast is encoded by *erg9* and utilizes farnesyl-pyrophosphate (FPP), a sesquiterpene precursor, and amorphadiene production is increased by knocking out the *erg9* gene, respectively. In another example discussing doxorubicin biosynthesis, multiple genes encoded by the *dxr* cluster were removed to improve desired protein production [167]. In addition, the efficiency of the heterologous system can be increased via the deletion of specific genes. NADPH-dependent enzymes are encoded by several natural pathways: for instance, oxidoreductases create metabolic pressure on the cell as the pathway metabolic flux gradually increases [167]. Chemler and coworkers [168] showed NADPH as a limiting factor in flavonoid (+)-catechins production in *E. coli*. In the study, gene knockouts were identified utilizing a metabolic modeling approach, for improving NADPH availability and thereby, flavonoid production [168]. Komatsu and coworkers [169] reported a ‘genome-minimized’ approach (deletion of non-essential elements) in *Streptomyces avermillitis*, in which the genome was reduced to 83% of its original size, creating space for the introduction of a gene cluster of streptomycin. The minimized genome of *Streptomyces* produced higher amounts of streptomycin, highlighting a prospective approach to enhance the production of biochemicals [169].

### 4.6. Metabolic Pathway Engineering

In this direction, the existence of divergence and homologies among genes between related metabolic pathways have led to switching genes and modules between interlinked pathways for novel microbial chassis [142]. The metabolic pathways of aromatic polyketide biosynthesis, namely, the macrolides, the teicoplanin, lipopeptides daptomycin/A54145, and aminocourmarins, highlight some examples. Hopwood and coworkers [170] used genes for related polyketides for combinational biosynthesis. In a key study, *Streptomyces* species (producing dihydrogranaticin and medermycin) were engineered by introducing actinorhodin pathway genes, resulting in hybrid antibiotics, dihydrogranatihordin, and mederrhodin, respectively [170]. For pathway engineering in a microbial system, genetic manipulation/switching biosynthetic genes downstream of the pathway defines higher success owing to the involvement of a few downstream enzymes [171]. The biosynthetic enzymes act on similar substrates/intermediates in closely related pathways. The creation of novel chimeras by gene switching showcases higher success potential and defines new research initiatives in the discovery and engineering of natural product pathways [142]. Novel fungal chimeras can be created via the reshuffling of genes and modules among linked pathways aimed at new combinations. Furthermore, a metabolic pathway can be engineered by altering a combination of genes in the biosynthetic pathway, for the creation of new chemical entities, subject to the tolerance of the downstream enzyme to substrate alteration [171]. One key concern in pathway engineering includes the non-disruption of the main scaffold, and the introduction of changes in the latter pathway steps has better chances of success, with the involvement of few downstream enzymes. Moreover, pathway engineering in the native host is carried out by new gene insertion/gene deletion or combinational pathway reconstitution [142].

### 4.7. CRISPR/Cas Genome Editing in Fungi

An emerging genome editing tool, CRISPR/Cas has witnessed key success in genome editing of filamentous fungi to produce high-value metabolites including pigments, enzymes, secondary metabolites, compounds of industrial importance, and agriculture, respectively. The CRISPR/Cas tool has been widely employed in improving fungal strains including *Aspergillus*, *Trichoderma*, and *Penicillium* sp. having industrial importance. Moreover, studies have documented the genetic manipulation of fungal strains for heterologous protein production. Manganese peroxidase, classified in the family of heme-containing peroxidases, degrades lignin and is produced by white-rot fungi, which has relevance in chemical industries. The two proteins, manganese peroxide and Interleukin 6, were produced in *Aspergillus* species [172]. Besides, socially important fungal strains, namely, *Mortierella alpinis* [173], *Fusarium veneratum* [174], *A. japonicas* [175], *Chrysosporium lucknowense* [176], have been developed for metabolites and protein production. Genetic manipulation strategies were attempted for *Cordyceps militaris* (edible medicinal mushroom) chassis; codon-optimized cas9 was used with promoter Pcmlsm3, and terminator Tcmura3 was expressed in the system. A CRISPR-Cas9 system comprising a single-strand DNA template, Cas9 DNA endonuclease, and RNA pre-synthesized in vitro was employed for insertion and site-specific deletion. The study aimed at genome editing of edible mushrooms for increasing genomic chassis and rapid development as ‘functional food’, respectively [177]. Chen and coworkers [178] employed CRISPR/Cas-mediated genome editing tools in *C. militaris* for enhanced ergothioneine production by discovering and regulating the metabolic pathway for ergothioneine biosynthesis [178]. In *Fusarium fujikuroi*, genetic manipulation methods were employed for enhanced gibberellic acid production [179].

### 4.8. Key Metabolic Engineering Studies in Fungi

Metabolic engineering of microbes has witnessed good translational success, with multiple bacterial and fungal species engineered for food additives production in recent times [180]. For the production of malic acid (used in food and beverages), overexpression of genes was attempted in *A. flavus*, *A. oryzae*, *S. cerevisiae*, etc. [181]. The CRISPR-Cas 9 engineering tool was employed to alter the molecular structure and colors of pigments, by introducing change in the desired sites. Another key study discussed the genetic manipulation of *Y. lipolytica* for β-hydroxylase and β-ketolase production by gene introduction in fungal species and increased astaxanthin production [182]. This study provided key inputs to produce astaxanthin, with a high commercial value. Research initiatives attempted in fungal chassis have substantially enhanced the production of high-value metabolites, thanks to contributions of transcriptome-based analysis, cloning, and mutational approaches in non-yielding species [77]. Moreover, emerging insights into different transcription stages and their manipulations have considerably increased the production of cellulases, amylases, and xylanases in filamentous fungi via gene overexpression [183].

## 5. Conclusions and Future Directions

Harnessing biological resources for high-value products in drug discovery and research is gaining considerable recognition; however, bottlenecks in low product yield in the native organism, tedious procedures in isolation and characterization, and limited knowledge of metabolic machinery make it an ardent task. Chemical modifications of natural scaffolds are challenging, and the major limiting factors have declined the interest in natural products, necessitating a need to address associated concerns. The bio-based production of high-value compounds highlights promising attributes, and recent advances in high-throughput technologies and synthetic biology have contributed immensely to addressing these limitations. Applications of state-of-the-art technologies including omics biology, computational approaches, genome analysis, and genome editing facilitate unprecedented outcomes.

Metabolic engineering approaches coupled with drug discovery and development can be a powerful tool, exemplified and discussed with significant examples of fungal chassis. Furthermore, emerging insights/knowledge of natural product pathways, their regulations, and dynamics have facilitated the screening and development of novel chemical entities, with the potential to the impact socio-economic arena. Significant translational success has been achieved in the genetic chassis of the biological organisms; however, it is difficult to predict the biosynthetic categories of both NPs and BGCs and the biological function, attributed to the complexities in the regulation of BGCs. Moreover, gene disruption methods have unraveled multiple biosynthetic pathways including the mycotoxin asirochlorine pathway [184], the protein phosphatase-2 inhibitor rubratoxin A pathway [185], and the mycotoxin cercosporin pathway [186]. In the industrial utilization of economically important fungal strains, a few limitations need to be addressed. For example, it is difficult to get high-density cultures due to pellet formation in the filamentous fungi; *Aspergillus* sp. and genetic engineering is cumbersome. Other limitations with high-throughput sequencing of fungal species with large colony formation and long process of mutant construction (a long time for spore formation in agar culture) further add to the limitations in industrial exploitation.

While significant advances in synthetic biology approaches have substantially contributed to the genetic manipulation of fungal organisms, challenges with precursor availability for natural product biosynthesis, low metabolic flux, and low enzyme activity in microbes add to the limitations [187]. In this direction, challenges concerned with the heterologous production of protein in filamentous fungi can be addressed by codon optimization and strong promoter selection at the transcription level, and other strategies, namely, the inclusion of N-glycosylation sites and signal sequences, and overexpression of chaperones, comprise methods to enhance protein production [188,189]. The heterologous production of terrequinone A and monacolin K polyketide was improved by the co-integration of BGC and universal regulator overexpression [190]. Metabolic engineering approaches individually or in combination, aided with the genomic tools, need to be developed further to address the existing concerns associated with the chassis of fungal organisms. The availability of limited marker genes necessitates the development of molecular genetic tools that would contribute to multidirectional improvement/developments in fungal biotechnology. CRISPR/Cas9-based genome editing has been quite successful; however, on genome-wide functional analysis, unwanted mutations are caused by microbial cells and they can be addressed by gene modification via homologous recombination [189], although a less feasible method. The application of newer technology, Target-AID, may circumvent this limitation in filamentous fungi. Metabolic engineering-mediated chassis of fungal organisms defines a prospective platform for obtaining high-value metabolites for socio-economic applications, subject to addressing the knowledge gaps/obstacles in fungal biology and engineering. 

## Data Availability

Not applicable.
